# Comparative transcriptome analysis of *Veratrum maackii* and *Veratrum nigrum* reveals multiple candidate genes involved in steroidal alkaloid biosynthesis

**DOI:** 10.1038/s41598-023-35429-5

**Published:** 2023-05-21

**Authors:** Dan Wang, Zhijing Yu, Meng Guan, Qinan Cai, Jia Wei, Pengda Ma, Zheyong Xue, Rui Ma, Kirsi-Marja Oksman-Caldentey, Heiko Rischer

**Affiliations:** 1 Jilin Provincial Key Laboratory of Agricultural Biotechnology, Jilin Academy of Agricultural Sciences, Changchun, 130033 Jilin Province People’s Republic of China; 2 VTT Technical Research Centre of Finland Ltd., P. O. Box 1000, 02044 VTT Espoo, Finland; 3 College of Life Sciences, Northwest A & F University, Yangling, 712100 People’s Republic of China; 4 Key Laboratory of Saline-Alkali Vegetation Ecology Restoration, Ministry of Education, College of Life Science, Northeast Forestry University, Hexing Road 26, Harbin, People’s Republic of China; 5 College of Agricultural Sciences, Yanbian University, Yanji, 133000 Jilin Province People’s Republic of China

**Keywords:** Gene expression, Transcriptomics, Secondary metabolism

## Abstract

*Veratrum* (Melanthiaceae; Liliales) is a genus of perennial herbs known for the production of unique bioactive steroidal alkaloids. However, the biosynthesis of these compounds is incompletely understood because many of the downstream enzymatic steps have yet to be resolved. RNA-Seq is a powerful method that can be used to identify candidate genes involved in metabolic pathways by comparing the transcriptomes of metabolically active tissues to controls lacking the pathway of interest. The root and leaf transcriptomes of wild *Veratrum maackii* and *Veratrum nigrum* plants were sequenced and 437,820 clean reads were assembled into 203,912 unigenes, 47.67% of which were annotated. We identified 235 differentially expressed unigenes potentially involved in the synthesis of steroidal alkaloids. Twenty unigenes, including new candidate cytochrome P450 monooxygenases and transcription factors, were selected for validation by quantitative real-time PCR. Most candidate genes were expressed at higher levels in roots than leaves but showed a consistent profile across both species. Among the 20 unigenes putatively involved in the synthesis of steroidal alkaloids, 14 were already known. We identified three new CYP450 candidates (CYP76A2, CYP76B6 and CYP76AH1) and three new transcription factor candidates (ERF1A, bHLH13 and bHLH66). We propose that ERF1A, CYP90G1-1 and CYP76AH1 are specifically involved in the key steps of steroidal alkaloid biosynthesis in *V. maackii* roots. Our data represent the first cross-species analysis of steroidal alkaloid biosynthesis in the genus *Veratrum* and indicate that the metabolic properties of *V. maackii* and *V. nigrum* are broadly conserved despite their distinct alkaloid profiles.

## Introduction

*Veratrum* is a genus comprising ~ 40 species of perennial herbaceous plants that grow widely in temperate regions of the northern hemisphere^1,2^. Although they are toxic, the roots of several *Veratrum* species are used, with caution, in the context of western herbalism and traditional Chinese medicine for the treatment of analgesia, inflammation^3,4^, tumors^5–7^, malignant ulcers^8^ and thrombosis^1^. The main active compounds are steroidal alkaloids such as jervine (and its derivative cyclopamine), cevanine and veratramine, which show a range of medically relevant bioactivities in cell-based assays and animal models^9,10^. The synthesis of steroidal alkaloids in *Veratrum* species requires isoprenoid units produced by the cytosolic mevalonate and MEP pathways, which are converted via several enzymatic steps into squalene, then the cyclic intermediate cycloartenol, and eventually cholesterol^11–13^. The conversion of cholesterol to steroidal alkaloids requires two major gene families: cytochrome P450 monooxygenases (CYP450s) and glycosyltransferases (GTs)^14–16^. Many of the enzymes involved in this downstream part of the pathway are unknown, as are the transcription factors that regulate steroidal alkaloid biosynthesis.

Comparative transcriptomics is a powerful approach that can identify genes encoding metabolic enzymes and the transcription factors that regulate them by comparing metabolically active tissues to controls lacking the pathway of interest^17,18^. The correlation of metabolite levels with RNA-Seq data recently identified four enzymes (including three CYP450s) involved in steroidal alkaloid biosynthesis in *Veratrum californicum*^19^. Furthermore, a comprehensive comparison of the stalk, leaf and root transcriptomes of *Veratrum nigrum* revealed 73 additional candidate genes involved in various parts of the pathway, including 11 enzymes in the segment from cycloartenol to cholesterol^10^. Here we applied a similar comparative transcriptomics approach, but we included two *Veratrum* species with different steroidal alkaloid profiles in order to probe for interspecific differences as well as common components of the *Veratrum* steroidal alkaloid biosynthesis pathways. We compared the *V. maackii* and *V. nigrum* root and leaf transcriptomes, and validated differences in gene expression by RT-PCR. Our results provide a valuable genomic resource for the discovery of new genes related to steroidal alkaloid metabolism.

## Results

### Differences in steroidal alkaloid profiles between *V. maackii* and *V. nigrum*

**Figure 1:**
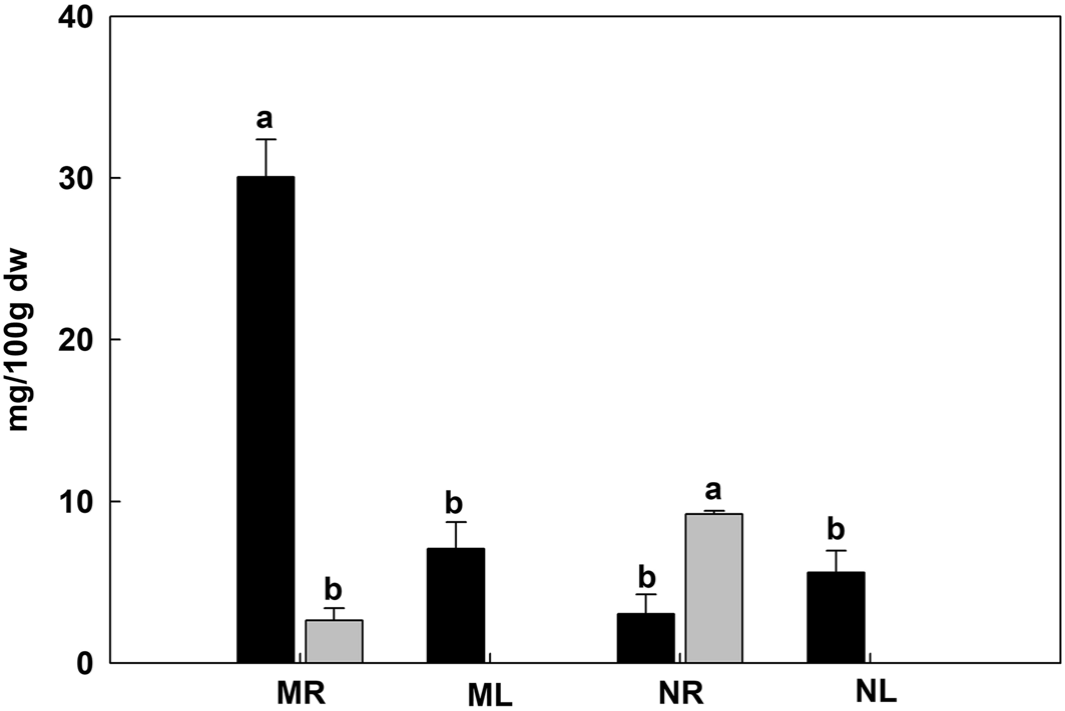
Steroidal alkaloid content of root and leaf tissues in *V. maackii* and *V. nigrum*. Data are means ± SD (n = 3). Black bar: jervine, gray bar: cyclopamine. Different lower case letters indicate significant differences (p < 0.05) according to Bonferroni multi-comparison tests. *dw* dry weight, *MR* *V. maackii* roots, *ML* *V. maackii* leaves, *NR* *V. nigrum* roots, *NL* *V. nigrum* leaves.

Previous RNA-Seq experiments focusing on *Veratrum* steroidal alkaloid biosynthesis have considered only individual species, therefore missing an opportunity to probe for interspecific differences as well as conserved aspects of the metabolic pathway^10,19^. To address this issue, we selected two *Veratrum* species (*V. maackii* and *V. nigrum*) with different alkaloid profiles, to maximize the likelihood of finding differences in underlying gene expression. We measured the content of jervine and cyclopamine in both plants (Fig. 1). Jervine was present in all samples but was significantly (p < 0.05) more abundant in *V. maackii* roots compared to *V. nigrum* roots, and compared to the leaves of both species. In contrast, cyclopamine was significantly (p < 0.05) more abundant in *V. nigrum* roots compared to *V. maackii* roots and compared to the leaves of both species (where cyclopamine was barely detected).

### De novo transcriptome assembly

Leaf and root tissues were collected from *V. nigrum* and *V. maackii* plants for RNA-Seq analysis (three biological replicates from different plants). We obtained 79.12 Gbp of clean reads from the 12 samples (Table 1). The GC content of each sample was at least 50.46%, and the Q30 base percentage was 92.23%. After processing with Trinity software, 437,820 transcripts were assembled with an N50 length of 871 bp, and 203,912 unigenes were identified with an average length of 687 bp and an N50 length of 780 bp.

**Table 1:** Properties of sequence data from *V. maackii* and *V. nigrum* samples.

Samples	Read number	Base number	GC content (%)	% ≥ Q30 (%)
MR1	21,438,133	6,398,015,619	50.85	93.71
MR2	22,031,394	6,579,776,547	51.15	92.57
MR3	21,995,374	6,566,543,213	51.33	93.09
ML1	19,173,584	5,724,322,855	50.46	93.26
ML2	18,070,632	5,397,563,767	50.70	92.23
ML3	21,033,939	6,280,572,696	50.97	93.16
NR1	25,975,986	7,756,946,919	50.60	92.54
NR2	24,366,903	7,276,845,424	50.56	93.49
NR3	24,590,721	7,343,721,697	51.11	93.66
NL1	21,886,237	6,533,703,658	51.58	92.60
NL2	19,334,525	5,771,620,207	51.19	93.55
NL3	25,089,517	7,486,197,094	51.61	93.08

MR1, MR2 and MR3 are three replicates of the root of *V. maackii*; ML1, ML2 and ML3 are three replicates of the leaves of *V. maacki*; NR1, NR2 and NR3 are three replicates of the root of *V. nigrum*; NL1, NL2 and NL3 are three replicates of the leaves of *V. nigrum.*

### Functional annotation

The 203,912 unigenes were compared with multiple public databases and 97,207 (47.67%) were annotated for all databases (Table 2).

**Table 2:** Summary of unigene annotations for the *V. maackii* and *V. nigrum* transcriptomes.

Annotated databases	Unigene	%
COG	27,902	28.70
GO	43,611	44.86
KEGG	21,581	22.20
KOG	54,907	56.48
Pfam	58,608	60.29
SWISS-PROT	48,803	50.20
TrEMBL	73,965	76.09
Nr	86,569	89.35
Nt	42,149	43.36

In the Nr database, 86,569 unigenes (42.45%) had significant matches and the homologous species were predicted. Accordingly, 10,329 (12%) and 9741 (11%) unigenes were aligned to the monocotyledonous plants *Elaeis guineensis* and *Phoenix dactylifera*, respectively (Supplementary Fig. S1A). For *V. nigrum*, 59,614 unigenes were annotated, 10,083 of which matched homologous sequences from *Elaeis guineensis*, followed by *Phoenix dactylifera* (9442) and *Musa acuminata* (3909) (Supplementary Fig. S1B). For *V. maackii*, 60,387 unigenes were annotated, 9618 of which matched homologous sequences from *Elaeis guineensis*, followed by *Phoenix dactylifera* (8926) and *Neonectria ditissima* (6434) (Supplementary Fig. S1C).

**Figure 2:**
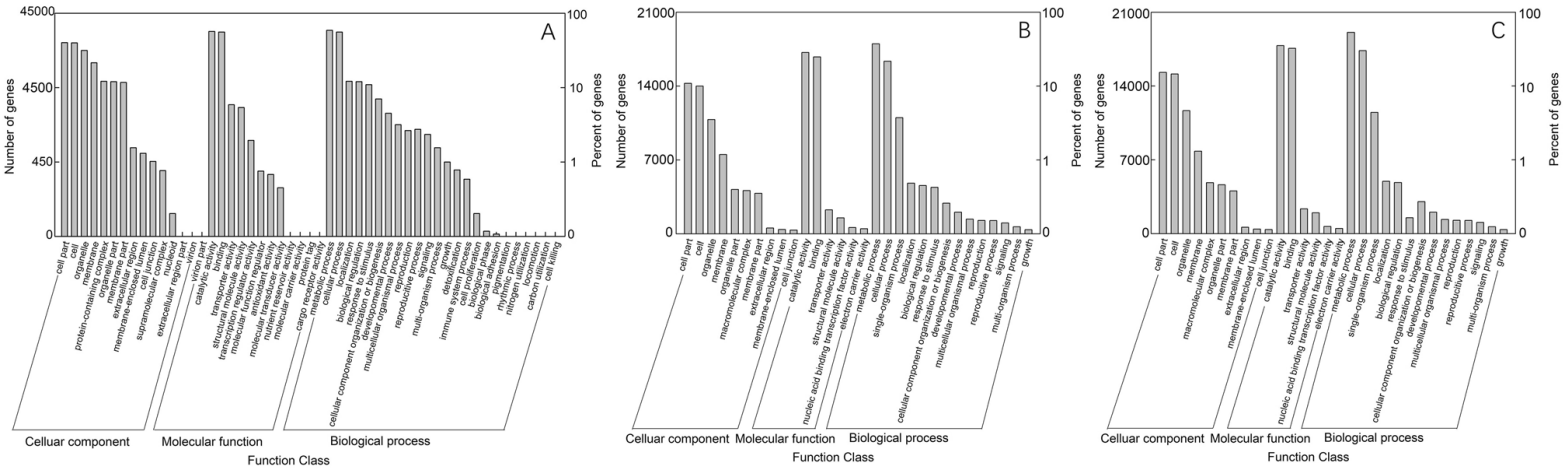
(**A**) GO classification of all assembled *V. maackii* and *V. nigrum* unigenes; (**B**) GO classification of all assembled *V. nigrum* unigenes; (**C**) GO classification of all assembled *V. maackii* unigenes.

Next, the 43,611 (44.86%) annotated unigenes were grouped into three Gene Ontology (GO) categories: biological process, cellular component and molecular function (Fig. 2A). In the biological process category, the three main subcategories were “metabolic process” (23,391, 31.26%), “cellular process” (20,911, 27.95%) and “localization” (6048, 8.08%). In the cellular component category, the three main subcategories were “cell part” (17,806, 23.34%), “cell” (17,662, 23.18%) and “organelle” (13,609, 17.79%). In the molecular function category, the three main subcategories were “catalytic activity” (22,517, 44.36%), “binding” (21,693, 42.73%), and “transporter activity” (2618, 5.16%). Separate GO annotations for the two species mirrored the overall annotation profile. For *V. nigrum*, 30,303 unigenes were annotated in the GO database, of which 22,263 were classified as biological process, 16,751 as cellular component and 24,764 as molecular function (Fig. 2B). For *V. maackii*, 31,254 unigenes were annotated in the GO database, of which 23,191 were classified as biological process, 17,524 as cellular component and 25,625 as molecular function (Fig. 2C).

**Figure 3:**
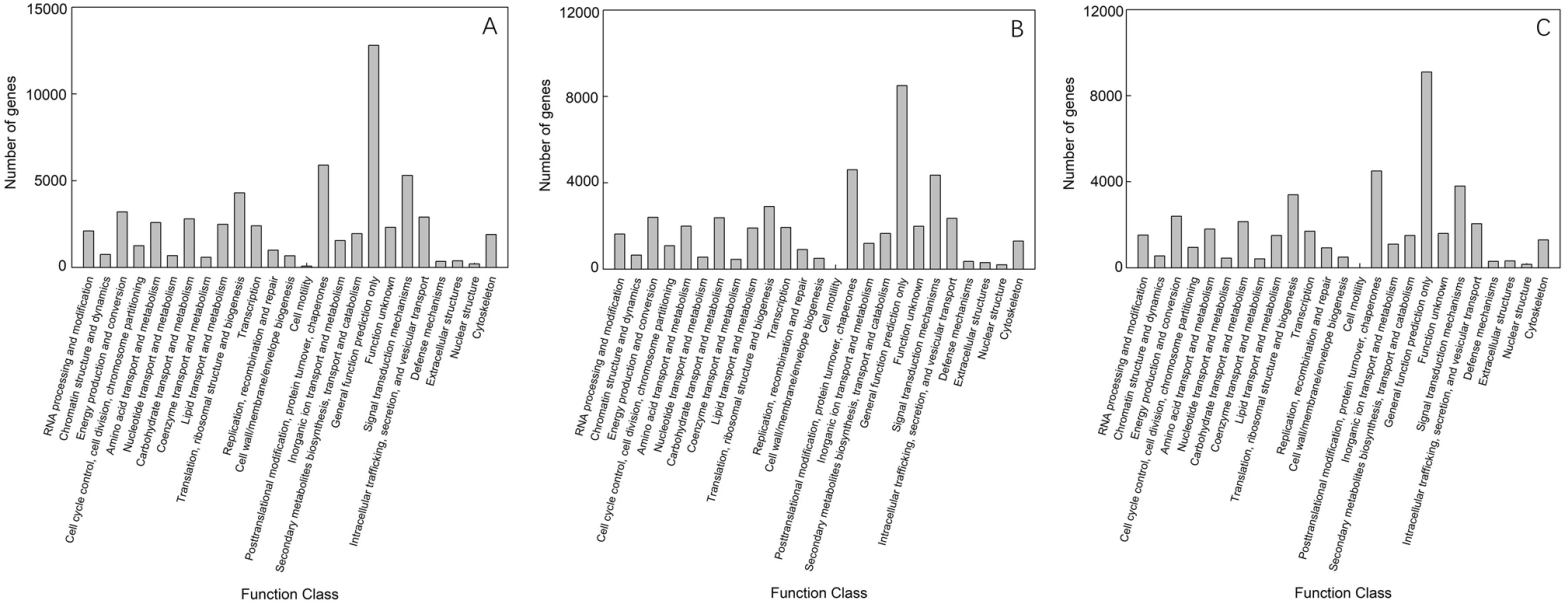
(**A**) KOG function classification of *V. maackii* and *V. nigrum* unigenes; (**B**) KOG function classification of *V. nigrum* unigenes; (**C**) KOG function classification of *V. maackii* unigenes.

In the KOG database, 54,907 (56.48%) unigenes were assigned to 26 categories. The most populated category (12,685 unigenes, 23.05%) was “general function prediction only”, followed by “posttranslational modification, protein turnover and chaperones” (6064, 11.04%), and “signal transduction mechanisms” (5317, 9.68%). The least populated categories were “cell motility” (41, 0.075%) and “nuclear structure” (268, 0.48%) (Fig. 3A). Again, the separate annotations for the two species mirrored the overall annotation profile. We identified 35,966 KOG annotations matching *V. nigrum* and the most populated category was “general function prediction only” with 8394 unigenes (Fig. 3B). We identified 37,395 KOG annotations matching *V. maackii* and the most populated category was “general function prediction only” with 9285 unigenes (Fig. 3C).

Finally, 21,581 unigenes were annotated against the KEGG database and mapped to 121 biological pathways (Supplementary Table S1). Among them, 6421 (29.75%) were involved in metabolic pathways, mainly including “ribosome” (1847, 8.5%), “purine metabolism” (698, 3.23%) and “glycolysis/gluconeogenesis” (666, 3.09%) (Supplementary Fig. S2A). Similar numbers of unigenes were annotated in each species: 14,456 in *V. nigrum* and 14,815 in *V. maackii*. In both species, the “metabolic pathways” category was most populated (3526 in *V. nigrum* and 3395 in *V. maackii*) followed by “biosynthesis of secondary metabolites” (1738 in *V. nigrum* and 1708 in *V. maackii*) (Supplementary Fig. S2B, C).

### Identification and analysis of differentially expressed genes

The transcriptomic comparison of two tissues in two species provides an opportunity to define the components of a tissue-specific metabolic pathway but also to evaluate differences in gene expression between species that lead to different metabolic profiles, which was not possible in earlier studies involving a single *Veratrum* species^10,19^. We found 3534 differentially expressed genes (DEGs) when comparing *V. maackii* roots vs *V. nigrum* roots (MR vs NR), 2004 upregulated and 1530 downregulated in *V. maackii*. Importantly 1019 transcripts were only present in MR and another 814 only in NR, indicating qualitative as well as quantitative differences in the cross-species transcriptomic comparison. We also found 5734 DEGs when comparing *V. maackii* leaves vs *V. nigrum* leaves (ML vs NL), 2919 upregulated and 2815 downregulated in *V. maackii*. As above, 1090 transcripts were only present in ML and another 902 only in NL.

**Figure 4:**
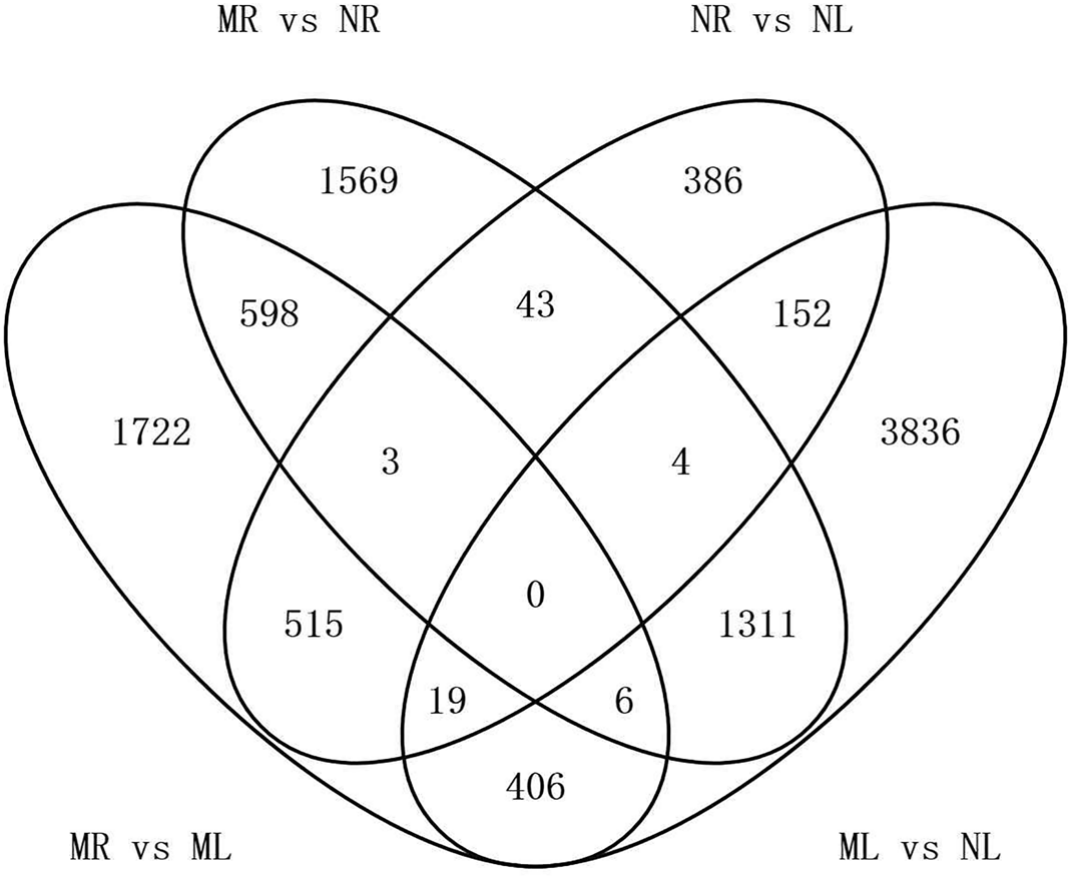
Venn diagram of differentially expressed genes in the roots and leaves of *V. maackii* and *V. nigrum*.

The within-species comparison of tissues revealed 3269 DEGs when comparing *V. maackii* roots and leaves (MR vs ML), 1593 upregulated and 1676 downregulated in the roots, including 862 transcripts that were only present in MR and another 339 only present in ML. Similarly, the NR vs NL comparison in *V. nigrum* revealed 1122 DEGs, 263 upregulated and 859 downregulated in the roots, including 75 transcripts that were only present in NR and 124 only present in ML (Fig. 4). Interestingly, zero transcripts were shared between all four sample types.

### Functional enrichment of differentially expressed genes

We examined the GO and KEGG annotations of the DEGs to determine any species-dependent or tissue-specific differences in gene functions. The GO biological process category “metabolic process” (GO:0008152) was more enriched in *V. maackii* than in *V. nigrum*. The GO biological process categories “oxidation–reduction process” (GO:0055114) and “rRNA processing” (GO:0006364), the GO cellular components “integral component of membrane” (GO:0016021), “membrane” (GO:0016020) and “chloroplast” (GO:0009507), and the molecular functions “ATP binding” (GO:0005524), “metal ion binding” (GO:0046872) and “oxidoreductase activity” (GO:0016491) were enriched in the leaves of both species but not in the roots. We observed major differences between leaves and roots in multiple GO categories (Supplementary Table S2).

Next, we identified the enriched KEGG pathways to focus on steroidal alkaloid metabolism (Supplementary Table S3). Approximately 100 pathways were enriched in the MR vs NR, ML vs NL and MR vs ML comparisons, but only 50 in NR vs NL. These involved 263, 329, 499 and 185 unigenes, respectively. In MR vs NR, “glycolysis/gluconeogenesis”, “purine metabolism” and “oxidative phosphorylation” were the most enriched pathways, each with 18 unigenes. In ML vs NL, “ribosome”, “purine metabolism” and “glycolysis/gluconeogenesis”, were the most enriched pathways, with 22, 20 and 19 unigenes, respectively. In MR vs ML, the most enriched categories were “photosynthesis”, “carbon fixation in photosynthetic organisms” and “phenylpropanoid biosynthesis”, with 58, 38 and 32 unigenes, respectively. Similarly, in NR vs NL, the most enriched categories were “photosynthesis”, “photosynthesis-antenna proteins” and “carbon fixation in photosynthetic organisms”, with 46, 24 and 22 unigenes, respectively. In the highly relevant category of “steroid biosynthesis”, the largest number of unigenes (five) was found in the MR vs ML comparison, whereas the differences in the MR vs NR and ML vs NL comparisons were insignificant (two unigenes). These data suggest there is only a small difference between species in terms of steroidal alkaloid metabolism but a large difference between leaf and root tissues.

### Identification of regulatory genes

**Figure 5:**
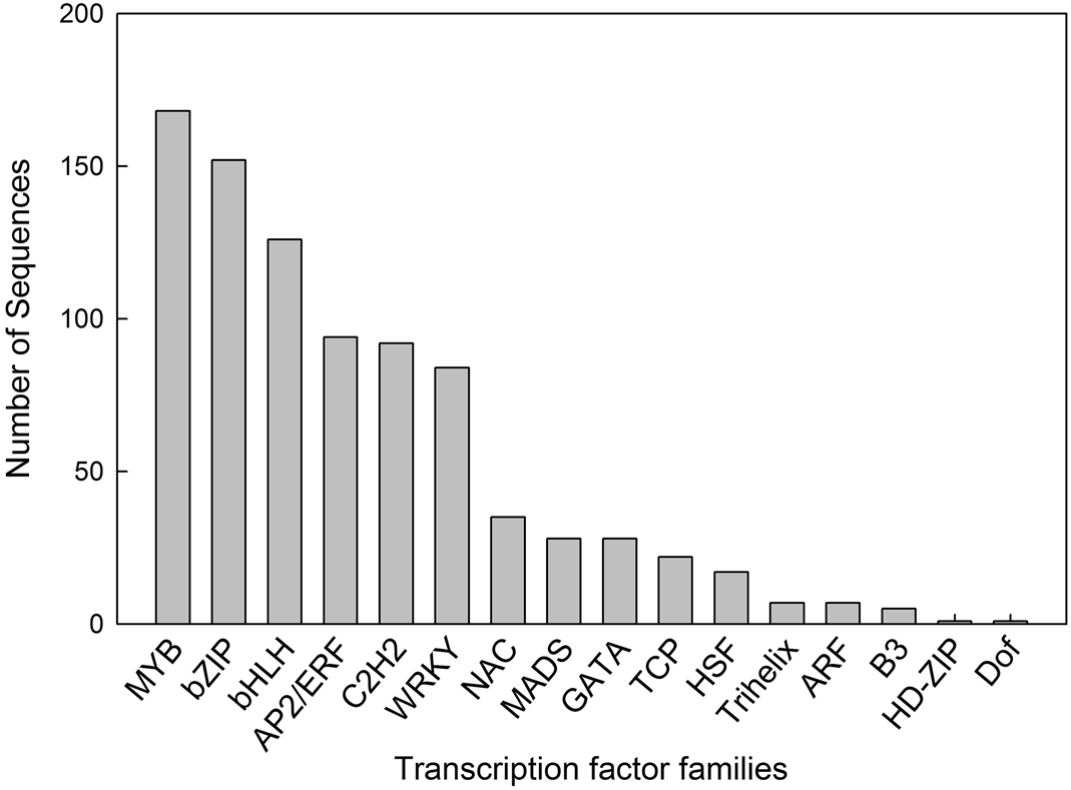
Distribution of sequences among *V. maackii* and *V. nigrum* transcription factor families.

One of the benefits of RNA-Seq analysis in the context of metabolic pathways is that differences in gene expression can identify not only enzymes involved directly in the metabolic pathway but also the regulatory proteins—especially transcription factors (TFs)—that control it. We were able to assign 867 unigenes to 16 known plant TF gene families, including 168 (19.36%) belonging to the MYB family, 152 (17.55%) to the bZIP family, 126 (14.56%) to the bHLH family, 94 (10.85%) to the ERF family and 92 (10.62%) to the C2H2 family (Fig. 5). Importantly, 234 of the TF genes were differentially expressed, including 131 when comparing MR vs ML and 37 when comparing NR vs NL, with the majority in both cases representing the MYB family (Supplementary Fig. S3). Another 42 TF genes differed when comparing MR vs NR, with the majority representing the C2H2 family, followed by MYB, AP2/ERF and bHLH. Furthermore, 77 TF genes differed when comparing ML vs NL, with the majority representing the zinc finger family, followed by bHLH, WRKY, NAC, MYB, ARF, GATA, bZIP and C2H2 (Supplementary Fig. S4). All differentially expressed bZIP, C2H2 and bHLH TF genes were upregulated in roots compared to the leaves in both species (Supplementary Fig. S4).

### Identification of CYP450 genes

We identified 444 unigenes annotated as CYP450s, 139 of which could not be assigned to a family. The rest were assigned to nine clans and 41 families (Supplementary Table S5; Supplementary Table S6). The most abundant families were CYP71 and CYP76, together accounting for more than half of the total, which is consistent with the distribution of CYPs found in other plant species^20^. We found that 153 of the CYP450 unigenes were differentially expressed, including the previously reported CYP90B27, CYP90G1 (two unigenes) and CYP94N that may be involved in cyclopamine synthesis^19^. The CYP450 unigenes were expressed at higher levels in the roots than the leaves in both species.

### Validation of candidate genes by RT-qPCR

**Figure 6:**
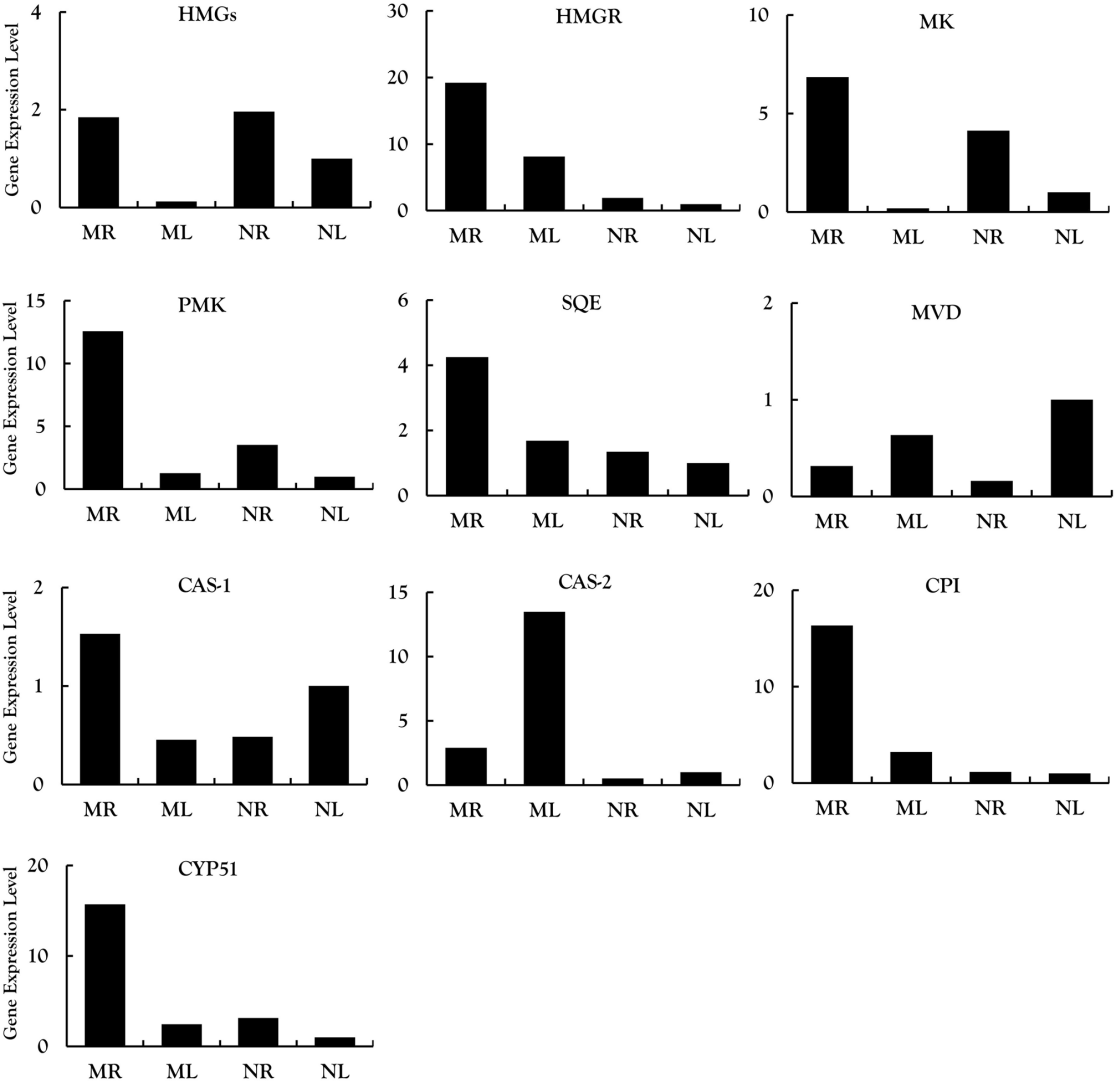
Analysis of 10 differentially expressed genes encoding enzymes involved in MVA and steroid alkaloid synthesis in the root and leaf tissues of *V. maackii* and *V. nigrum* validated by qRT-PCR.

**Figure 7:**
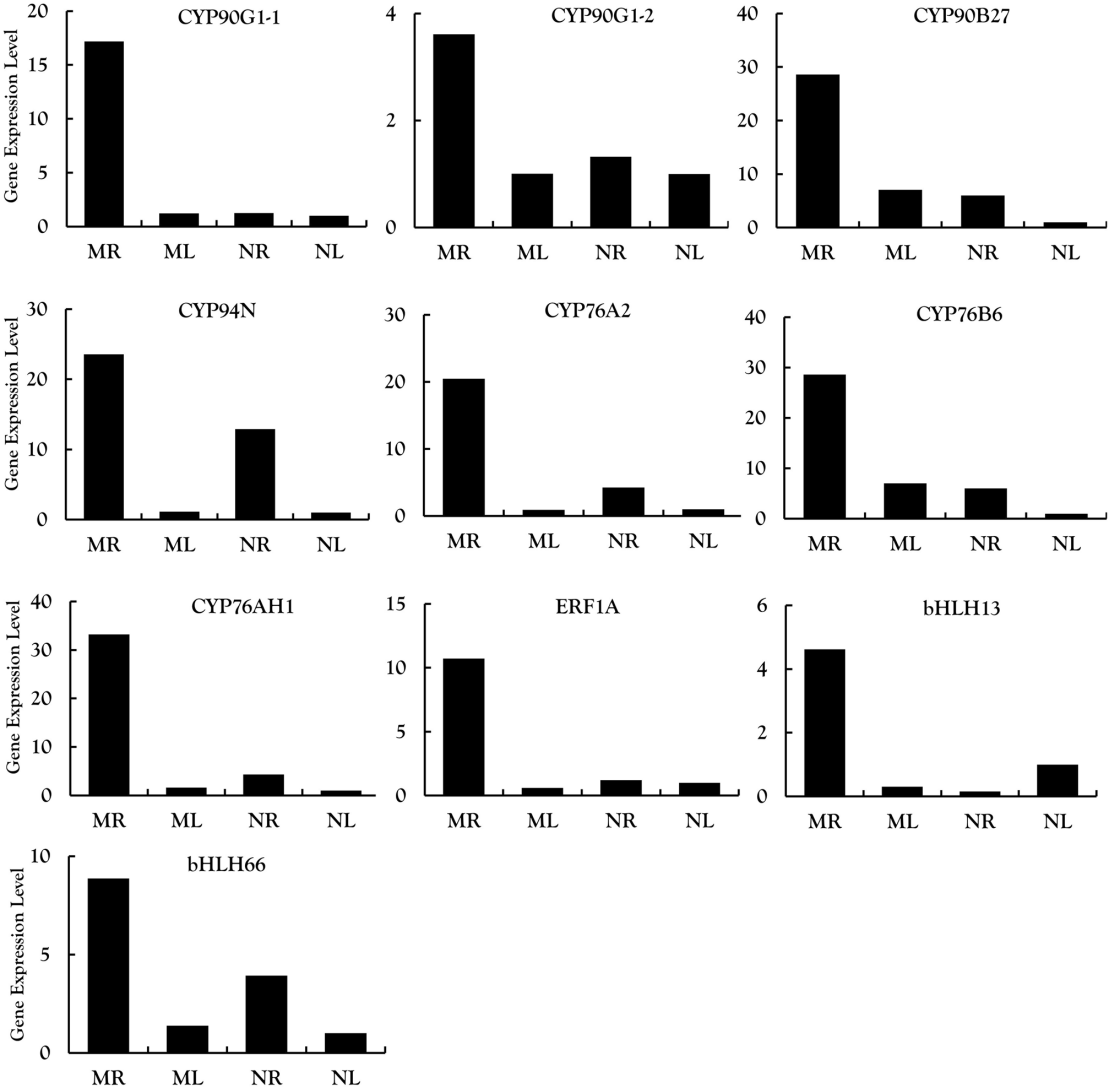
Analysis of seven CYP50 genes and three transcription factors potentially involved in steroid alkaloid synthesis validated by qRT-PCR.

We selected 20 candidate genes from among the DEGs encoding enzymes and TFs associated with the steroidal alkaloid pathway and validated their expression profiles by RT-qPCR. All 10 candidate genes encoding enzymes in the upstream part of the steroidal alkaloid pathway were expressed at higher levels in the roots than the leaves of both species (Fig. 6): 3-hydroxy-3-methylglutaryl-CoA synthase (HMGS), 3-hydroxy-3-methylglutaryl-CoA reductase (HMGR), mevalonate kinase (MK), diphosphomevalonate kinase (PMK), diphosphomevalonate decarboxylase (MVD), squalene epoxidase (SQE), cycloartenol synthase (CAS, two unigenes), cyclopropyl isomerase (CPI) and sterol 14-demethylase (CYP51). Similarly, all seven CYP450 candidate genes were expressed more strongly in roots than leaves: CYP90G1 (two unigenes), CYP90B27, CYP94N, CYP76A2, CYP76B6 and CYP76AH1 (Fig. 7). Finally, among the three TF genes we tested, ERF1A and bHLH66 were expressed more strongly in the roots whereas bHLH13 was expressed more strongly in the leaves, suggesting the latter may be a negative regulator (Fig. 7). The RT-qPCR results were consistent with the RNA-Seq data for most candidate genes, except that MVD was expressed at a higher level in NL than NR and CAS2 was expressed at a higher level in ML than MR.

## Discussion

### Differential accumulation of steroidal alkaloids

*Veratrum* plants produce a rich variety of steroidal alkaloids many of which are of pharmaceutical interest, and the alkaloid profiles of different species are unique. For example, the steroidal alkaloid profile of *V. californicum* includes the significant accumulation of cyclopamine in rhizomes, followed by roots and bulbs, resulting in a 20-fold difference between underground and aerial organs^19^. This suggests that steroidal alkaloids in *V. californicum* are synthesized in underground organs and transported to other parts of the plant. The comparison of *V. californicum* roots and leaves indicated a 20-fold to 100-fold higher content of cyclopamine in the roots^21,22^, but this varies considerably with harvest location and growth stage^22^. Similarly, *V. nigrum* roots were found to accumulate twice as much jervine as the leaves^23^. As the basis for our RNA-Seq experiments, we extended this analysis to include multiple alkaloids and multiple species. We found that *V. nigrum* and *V. maackii* showed opposing profiles for the accumulation of jervine and cyclopamine, with jervine accumulating to much higher levels in *V. maackii* roots compared to leaves and to the roots and leaves of *V. nigrum*, whereas cyclopamine was more abundant in the roots of *V. nigrum* compared to *V. maackii* and was not detected in the leaves of either species. These results show that *V. nigrum* and *V. maackii* are good models for the analysis of interspecific differences in alkaloid accumulation, which could be due to various factors including differential gene expression, protein synthesis/stability, enzyme activity and or product turnover. As mentioned above, the accumulation of alkaloids is influenced by environmental factors and varies with growth stage and season. In most studies the harvesting season of the studied material is not mentioned but our study confirms the pattern observed for cyclopamine in *V. californicum* where the underground organs generally accumulate higher alkaloid concentrations than aboveground organs^19,22^. For jervine this is true for *V. maackii* but for *V. nigrum* the ratio is more balanced. There seems to be a tendency to accumulate more alkaloids towards fall in temperate *Veratrum* species, but our data are based on a single collection in early spring representing a snapshot only and information on the dynamics of alkaloid accumulation in *V. maackii* and *V. nigrum* are missing. Specific environmental cues leading to stimulation of alkaloid biosynthesis are unknown but master regulators such as the identified transcription factors likely play a central role in the signalling cascade.

### De novo assembly and gene functional classification

We assembled separate transcriptome libraries for root and leaf tissue in *V. nigrum* and *V. maackii* to facilitate comparative transcriptomic analysis of tissue-specific and species-dependent gene expression in the context of steroidal alkaloid biosynthesis. This extends the analysis of *V. nigrum* by Szeliga et al.^10^ although they considered three separate tissues (root, leaf and stalk). The inclusion of two aerial tissues refines the analysis but the main relevant distinctions resulting from their work was between the roots and other tissues, so we restricted our analysis to roots vs leaves in order to reduce the number of transcriptome datasets. This simplification allowed us to construct and analyze larger transcriptome libraries compared to the Szeliga study, leading to the isolation of 132,155 and 109,287 unigenes in the *V. nigrum* root and leaf, respectively (Supplementary Fig. S5A), as well as 124,802 and 101,035 in the *V. maackii* root and leaf, respectively (Supplementary Fig. S5B). In NR, NL, MR and ML, we found 43,608, 20,740, 43,927 and 20,160 unigenes, respectively (Supplementary Fig. S5A,B).

Szeliga and colleagues annotated their transcriptome datasets and found more annotated unigenes in the leaves than the roots^10^. In contrast, we found more annotated unigenes in the root transcriptomes of both species) (Supplementary Table S7). Both studies found the largest numbers of annotations in the Nr database. We were able to annotate 64,329 (66%) unigenes in MR, 43,151 (44%) in ML, 61,428 (63%) in NR and 46,139 (47%) in NL. We also identified more unigenes that could be annotated in KEGG (21,581). The differences between our results and those in the earlier study of *V. nigrum* may reflect the different sources of plants (wild plants in our study, but laboratory-grown plants in the earlier study^10^) and/or differences in the timing of plant collection for sampling.

Maximally 12% of the unigenes had homologous matches with individual other species. This value is too low to substantiate close phylogenetic relationship. Relationship within monocotyledons is however clearly indicated. Crop plants are generally well represented in databases and therefore the best hits, *Elaeis guineensis* and *Phoenix dactylifera*, should not be overrated. A previous study on *V. nigrum*^10^ notes alignments with *Oryza sativa*, a monocotyledon crop, which is lacking steroidal alkaloids, too.

### Genes encoding enzymes involved in steroidal alkaloid biosynthesis

**Figure 8:**
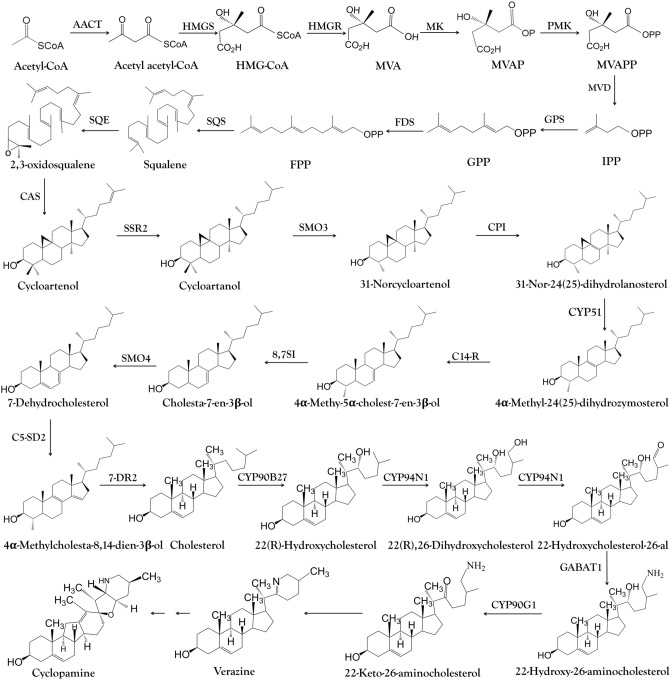
Cyclopamine biosynthesis pathway. *AACT* acetyl-CoA C-acetyltransferase, *Acetoacetyl-CoA* acetoacetyl coenzyme A, *Acetyl-CoA* acetyl coenzyme A, HMGCoA:3-hydroxy-3-methylglutaryl CoA, *HMGS* hydroxymethylglutaryl-CoA synthase, *HMGR* 3-hydroxy-3-methylglutaryl CoA reductase, *MVA* mevalonate, *MK* mevalonate kinase, *MVAP* mevalonate-5-phosphate, *PMK* phosphomevalonate kinase, *MVD* diphosphomevalonate decarboxylase, *MVAPP* mevalonate-5-diphosphomevalonate, *IPP* isopentenyl diphosphate, *GPS* geranylgeranyl diphosphate synthase, *GPP* geranyldiphosphate, *FDS* farnesyl diphosphate synthase, *FPP* farnesyldiphosphate, *SQS* squalene synthetase, *SQE* squalene epoxidase, *CAS* cycloartenol synthase, *SSR2* sterol side chain reductase 2, *SMO3* C-4 sterol methyl oxidase 3, *CPI* cyclopropylsterol isomerase, *CYP51* sterol C-14 demethylase, *C14-R* sterol C-14 reductase, *CYP51* sterol C-14 demethylase, *SMO4* C-4 sterol methyl oxidase 4, *C5-SD 2* sterol C-5(6) desaturase 2, *7-DR2* 7-dehydrocholesterol reductase 2, *CYP90B27* cholesterol 22-hydroxylase, *CYP94N1* 22-hydroxycholesterol 26-hydroxylase/oxidase, *GABAT1* 22-hydroxycholesterol-26-al transaminase, *CYP90G1* 22-hydroxy-26-aminocholesterol 22-oxidase.

The biosynthesis of steroidal alkaloids can be divided into three stages: the upstream MVA pathway that produces 2,3-oxidosqualene, the conversion of cycloartenol to cholesterol, and the conversion of cholesterol to steroidal alkaloids (Fig. 8)^24,25^. The first stage involves six key enzymes (HMGS, HMGR, MK, PMK, MVD and SQE) and we screened the corresponding genes, which were also identified by Szeliga et al.^10^. We found that most of these genes were strongly expressed in roots, with MVD as the only exception (Fig. 6). HMGR is a rate-limiting step in the MVA pathway, catalyzing the transformation of 3-hydroxy-3-methylglutaryl-coenzyme A (HMGR-CoA) into mevalonate (MVA)^26^. HMGR was expressed more strongly in roots than leaves in both species, as confirmed herein and previously^10^ by qRT-PCR. The second stage of steroidal alkaloid synthesis involves three genes encoding the enzymes CAS, CPI and CYP51. We found that CPI and CYP51 were expressed at higher levels in the roots of both species than the leaves (Fig. 6). CPI catalyzes the conversion of 31-norcycloartanol to 31-nor-24(25) dihydrolanosterol and CYP51 catalyzes 14α-demethylation during the initial reaction of plant sterol biosynthesis^27^. Both CPI and CYP51 are involved in cholesterol biosynthesis in solanaceous plants^25^. Importantly, cycloartenol is considered the first cyclic intermediate in the biosynthesis of steroidal alkaloids but an alternative pathway has been proposed in *Arabidopsis thaliana* involving the conversion of 2,3-oxidosqualene into lanosterol. The absence of annotated transcripts encoding lanosterol synthase in our dataset suggests that this alternative pathway is not present or is present but silenced in *V. nigrum* and *V. maackii*, confirming that steroidal alkaloids are synthesized from cycloartenol in these species^25,28^.

## Genes encoding transcription factors involved in steroidal alkaloid biosynthesis

To identify TFs that regulate metabolism in *V. maackii* and *V. nigrum*^29^, we screened annotated unigenes representing different TF families and found that the most abundant were MYB, bZIP, bHLH, AP2/ERF and C2H2. The ranking differed slightly in the two species (*V. maackii*—MYB, C2H2, bZIP, bHLH, WRKY, NAC and AP2/ERF; *V. nigrum*—MYB, bZIP, WRKY, C2H2, AP2/ERF, GATA and bHLH) and also differed from the results provided by Szeliga and colleagues (bHLH, C2H2, HD-ZIP, MYB and C3H), where the bHLH family was found only in stalk and root tissues while the C2H2 family was only found in the leaves^10^. In contrast, we found that the bHLH and C2H2 families were expressed in roots and leaves and were upregulated in roots compared to leaves. This discrepancy may reflect the differences in plant sources discussed above and/or the larger number of unigenes discovered in our study.

We selected three TF genes for validation by qRT-PCR. The first was AP2/ERF, which was expressed at significantly higher levels in the roots than the leaves of both species and was also expressed at significantly higher levels in *V. maackii* compared to *V. nigrum* roots (MR vs NR) whereas there was a nonsignificant interspecific difference in leaves (ML vs NL). The AP2/ERF protein GAME9 is a TF in solanaceous plants that can interact with SIMYC2 to regulate the expression of upstream genes such as C5-SD (encoding D(7)-sterol-C5(6)-destaurase) to regulate the synthesis of glycoside alkaloids^30^. It is possible that AP2/ERF plays a similar role in *Veratrum* plants. The strong correlation between ERF1A expression and the cyclopamine content of roots and leaves in both species suggests that ERF1A specifically regulates the synthesis of cyclopamine in *V. maackii* roots. The other two candidates are members of the bHLH family, which is known to regulate the synthesis of flavonoids and alkaloids^31^. We found that bHLH13 and bHLH66 were most strongly upregulated in *V. maackii* roots, and that bHLH66 was significantly (p < 0.05) more abundant in the roots of both species than the leaves, but especially in *V. maackii* (Fig. 7). We speculate that both TFs may regulate steroidal alkaloid biosynthesis. The strong correlation between expression of these two TFs and the steroidal alkaloid content indicated that bHLH13 might regulate cyclopamine synthesis whereas bHLH66 might regulate the conversion of cyclopamine to jervine. Few previous studies have addressed the transcriptional regulation of steroidal alkaloid biosynthesis in *V. nigrum*^10,19^ and none (to our knowledge) in *V. maackii*, so these three candidates should be examined in more detail in future studies to determine their precise roles.

### Candidate CYP450s involved in steroidal alkaloid biosynthesis

The CYP450 family is the largest enzyme family in plant metabolism and it plays a key role in the diversification and functional modification of triterpenoid and sterol skeletons^32,33^. We examined seven differentially expressed CYP450 genes that may be involved in steroid alkaloid biosynthesis. Three of them (CYP90B27, CYP90G1 and CYP94N1) catalyze the conversion of cholesterol into downstream intermediates. CYP90B27 converts cholesterol to 22(R)-hydroxylcholesterol, which is oxidized in two steps to form 22-hydroxylcholesterol-26-al by CYP94N1. Another enzyme (GABAT1) converts 22-hydroxylcholesterol-26-al into 22-hydroxyl-26-aminolcholesterol, which is in turn converted into 22-keto-26-aminolcholesterol by CYP90G1 (Fig. 8). We found that the transcripts for CYP90G1, CYP94N and CYP90B27 were significantly (p < 0.05) more abundant in *V. maackii* and *V. nigrum* roots than leaves (Fig. 7), which is consistent with earlier reports^19^. The strong correlation between CYP90G1-1 expression and the steroidal alkaloid content of the tissues in both species indicated that CYP90G1-1 might be the key enzyme for steroidal alkaloid biosynthesis in *V. maackii* roots (Figs. 1 and 7). The root is the main medicinal part of the *Veratrum* plant due to the accumulation of steroidal alkaloids^34^. It is therefore anticipated that enzymes responsible for the decoration of the alkaloid skeleton would be predominantly expressed in the roots. Three further CYP450 unigenes (CYP76A2, CYP76B6 and CYP76AH1), with strong expression in the roots according to RNA-Seq data, were validated by qRT-PCR (Fig. 7).

**Figure 9:**
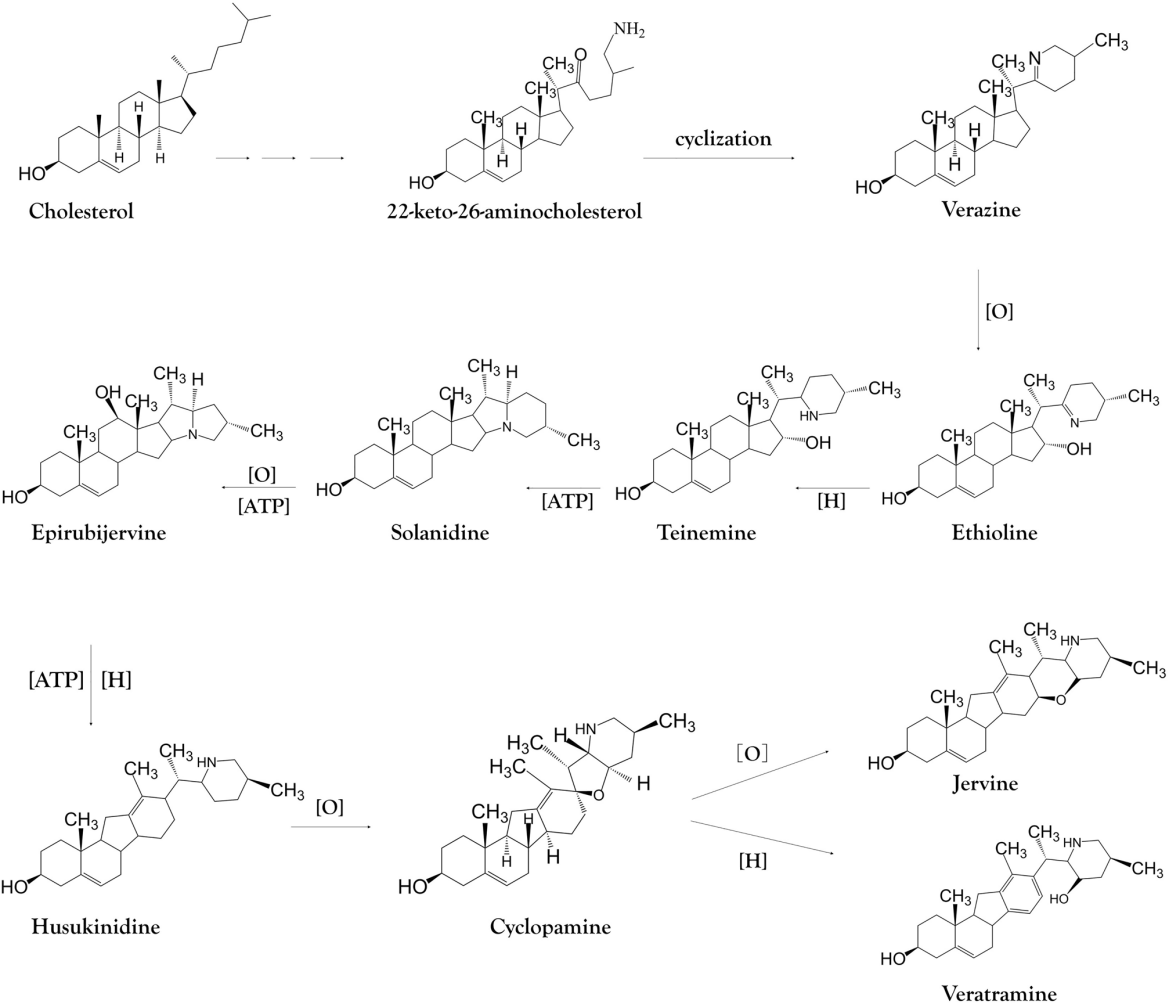
Proposed pathway for the conversion of 22-keto-26-aminocholesterol to cyclopamine in *Veratrum* plants.

In the third stage of steroidal alkaloid synthesis, the enzymes that convert 22-keto-26-aminocholesterol to cyclopamine are generally unknown (Fig. 9). Verazine, the predicted precursor of cyclopamine, is most likely formed by the spontaneous cyclization of 22-keto-26-aminocholesterol, a reactive intermediate. Verazine possibly undergoes 16a-hydroxylation to form ethioline catalyzed by 16DOX and is then reduced to form teinemine and is converted to solanidine in the presence of light. Solanidine is oxidized to form epirubijervine and is reduced in the presence of light to form husukinidine. The latter is then transformed into cyclopamine and further into jervine by additional oxidation reactions, or is reduced to form veratramine^11,35–41^. Given the correlation between CYP450 expression and the distribution of steroidal alkaloids, we speculate that these enzymes are also involved in the final part of steroidal alkaloid biosynthesis pathway. The strong correlation between CYP76AH1 expression and the steroidal alkaloid content of the tissues in both species suggests that CYP76AH1 is specifically involved in the final segment of steroidal alkaloid biosynthesis in *V. maackii* roots (Figs. 1 and 7). The precise functions of the three new candidate genes CYP76A2, CYP76B6 and CYP76AH1 in the final part of the steroidal alkaloid biosynthesis pathway should be evaluated in more detail.

In conclusion, we combined RNA-Seq analysis and the quantification of specific steroidal alkaloids in two tissues from two *Veratrum* species to facilitate the rapid identification of genes encoding enzymes and transcription factors related to secondary metabolism in a genus of non-model medicinal plants. We identified 97,207 V*. nigrum* and *V. maackii* unigenes which were annotated using the Nr, GO, KOG and KECG databases, including 3534 genes that were differentially expressed between tissues or between species. We found that 235 differentially expressed unigenes encoded enzymes with a potential role in steroidal alkaloid biosynthesis. Twenty unigenes putatively involved in steroidal alkaloid synthesis, including 14 known genes, three new candidate genes encoding CYP450s (CYP76A2, CYP76B6 and CYP76AH1), and three new candidate genes encoding TFs (ERF1A, bHLH13 and bHLH66), were rapidly identified by comparative transcriptome analysis. We propose that ERF1A, CYP90G1-1 and CYP76AH1 are specifically involved in the key steps of steroidal alkaloid biosynthesis in *V. maackii* roots. Our data provide a valuable reference for the study of steroidal alkaloid biosynthetic pathways across *Veratrum* species, helping to identify the genes involved in the main biosynthetic pathway and also those responsible for the species-dependent steroidal alkaloid profiles.

## Materials and methods

### Plant material

**Figure 10:**
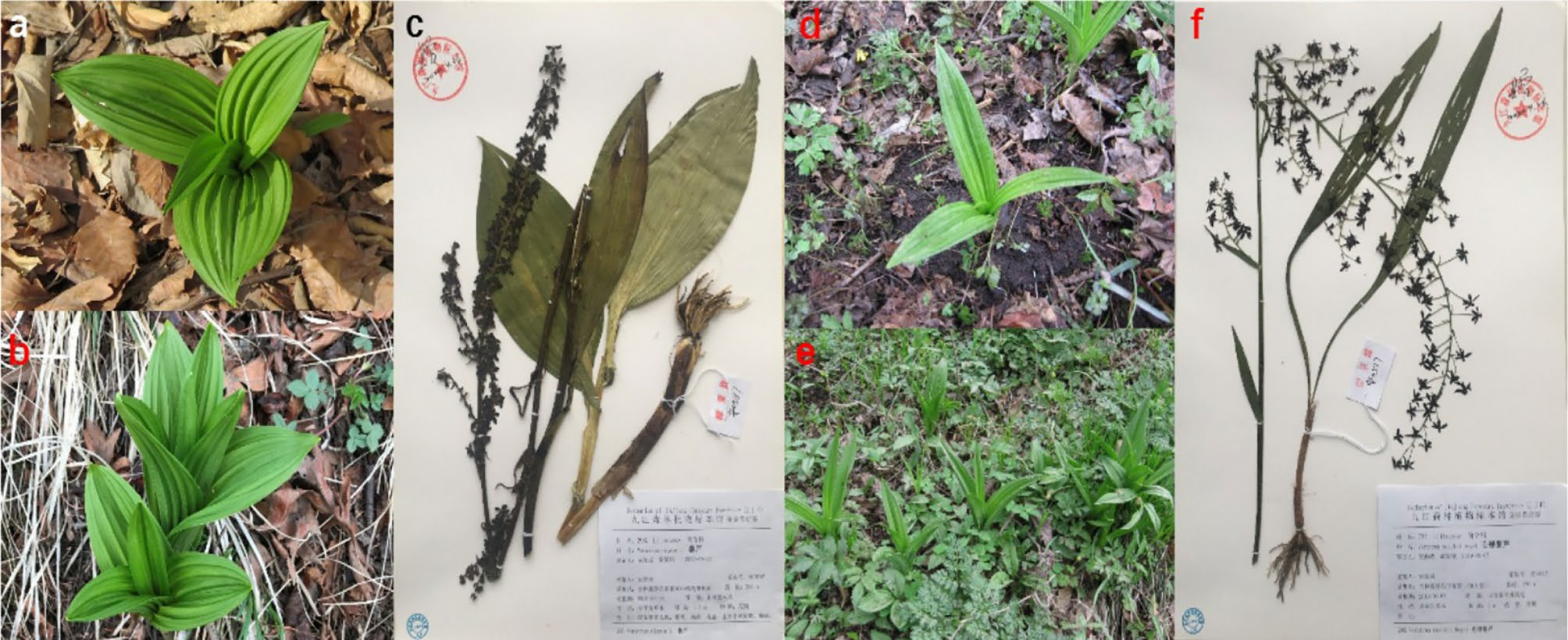
*Veratrum nigrum* and *Veratrum maackii* collected on Changbai Mountain in the Jilin Province of China. (**a**–**c**) *Veratrum nigrum* plants; (**d**–**f**) *Veratrum maackii* plants. Vouchers were deposited in Herbarium of Jiujiang Forestry Institute, Jiangxi Province, China (Numbers: AN0517, AN0587).

Wild *V. nigrum* and *V. maackii* plants were collected on Changbai Mountain in the Jilin Province of China in April 2018 in accordance with “Regulations of the State on Wild Plant Protection (No 687, 2017)”. The species were confirmed by a botanist (An Haicheng) and the vouchers were deposited in the Herbarium of Jiujiang Forestry Institute, Jiangxi Province, P. R. China (Numbers: AN0517, AN0587) (Fig. 10). Roots and leaves were used for steroidal alkaloid analysis and transcriptome sequencing. Plant samples were frozen in liquid nitrogen and then stored at – 80 °C before RNA isolation. The corresponding samples were lyophilized and powdered for steroidal alkaloid extraction.

### Determination of steroidal alkaloid content

We soaked 10 g of lyophilized and powdered sample in 40 ml of 80% ethanol at room temperature for 1 h, and then in 95% ethanol (1:4 w/v) at 80 °C for 2 h with continuous stirring. The solvent was collected by filtration. The sample residue was extracted again in 95% ethanol (1:10 w/v) under the same conditions. This procedure was repeated a third time. The three filtrates were combined, the ethanol was removed by evaporation, and then the extract was dissolved in methanol for subsequent analysis. The concentrations of jervine and cyclopamine in the extract were determined by HPLC–UV using commercial cyclopamine and jervine (LC Laboratories, Woburn, MA, USA) as reference compounds^42^.

All samples were analysed in triplicates. SigmaPlot 10.0 (Systat Software Inc., San Jose, USA) was used for statistical analysis and plotting. Significant differences in alkaloid contents within species' root and leaf tissues were identified by ANOVA. Multi-range comparisons were performed using Bonferroni multi-comparison tests and different lowercase letters indicate significant differences (p < 0.05).

### RNA extraction, library construction and transcriptome sequencing

Total RNA was extracted from the 12 samples using Trizol Reagent. RNA quality and quantity were assessed using a NanoPhotometer spectrophotometer, and RNA integrity was examined by 1% agarose gel electrophoresis. Library construction and RNA-Seq analysis were carried out by Shanxi Boride Biotechnology using the Illumina HiSeq 2500 platform. Qubit2.0 and Agilent 2100 were used to determine the library concentration and insert size, and qPCR was used for library quantification and quality control. Trinity was used to assemble the transcript sequences.

### Functional annotation of unigenes

BLAST (E-value < 1 × 10^–5^)^43^ was used to align unigenes against multiple protein and nucleotide databases: Nr (RefSeq non-redundant proteins)^44^, GO (Gene Ontology)^45^, COG (Clusters of Orthologous Groups)^46^, KOG (euKaryotic Orthologous Groups)^47^, KEGG (Kyoto Encyclopedia of Genes and Genomes)^48^, Pfam (Protein family)^49^, TrEMBL^50^ and SWISS-PROT^51^.

### DEG analysis

DEGs were identified using DESeq^52^. The false discovery rate (FDR) was used to determine the p value threshold. A FDR < 0.01 and a fold change (FC) ≥ 2 were applied as screening criteria to identify DEGs between pairs of samples.

### Validation of gene expression by RT-qsPCR analysis

Total RNA was reverse transcribed into cDNA using the Integrated First Strand cDNA Synthesis Kit (DiNing, Beijing, China). The expression levels of unigenes putatively involved in sterol synthesis were monitored by RT-qPCR using the primer sequences listed in Table S1 (Supplementary Table S8). RT-qPCR analysis was performed with a Roche LightCycler480 Real-time PCR System using SYBR Premix Ex Taq (TaKaRa, Tokyo, Japan). The reaction conditions were as follows: 95 °C for 10 min, and 40 cycles of 95 °C for 15 s, 58 °C for 1 min. The actin (ACT) gene was used as an internal reference, and the relative expression level of each unigene was determined using the 2−ΔΔCt method^53^.

### Sample collection

Samples were collected by an accredited botanist (An Haicheng) in accordance with “Regulations of the State on Wild Plant Protection (No 687, 2017)” and the vouchers were deposited in the Herbarium of Jiujiang Forestry Institute, Jiangxi Province, P. R. China (Numbers: AN0517, AN0587).

## Supplementary Information

Supplementary Figures.

Supplementary Table S1.

Supplementary Table S2.

Supplementary Table S3.

Supplementary Table S4.

Supplementary Table S5.

Supplementary Table S6.

Supplementary Table S7.

Supplementary Table S8.

## Data Availability

All the essential data associated with this manuscript are made available as supplementary data and in online repositories at https://www.ncbi.nlm.nih.gov/, PRJNA912756.
