# Supplementary material for: Comparative transcriptome analysis of Veratrum maackii and Veratrum nigrum reveals multiple candidate genes involved in steroidal alkaloid biosynthesis
Source: Sci Rep. 2023 May 21;13:8198. doi: 10.1038/s41598-023-35429-5 (PMC10200796; doi:10.1038/s41598-023-35429-5)

Comparative analysis of the leaf and root transcriptomes in *Veratrum maackii* and *Veratrum nigrum* reveals multiple candidate genes involved in steroidal alkaloid biosynthesis

Dan Wang[1, 5†,] Zhijing Yu[1†], Meng Guan [4†], Qinan Cai[1], Jia Wei[1], Pengda Ma[3], Zheyong Xue[4], Rui Ma[1*], Kirsi-Marja Oksman-Caldentey[2], Heiko Rischer[2*]

Supplementary files

Supplementary Figure S1A. Distribution of homologous species in the Nr database matching unigenes from *V. maackii* and *V. nigrum*.

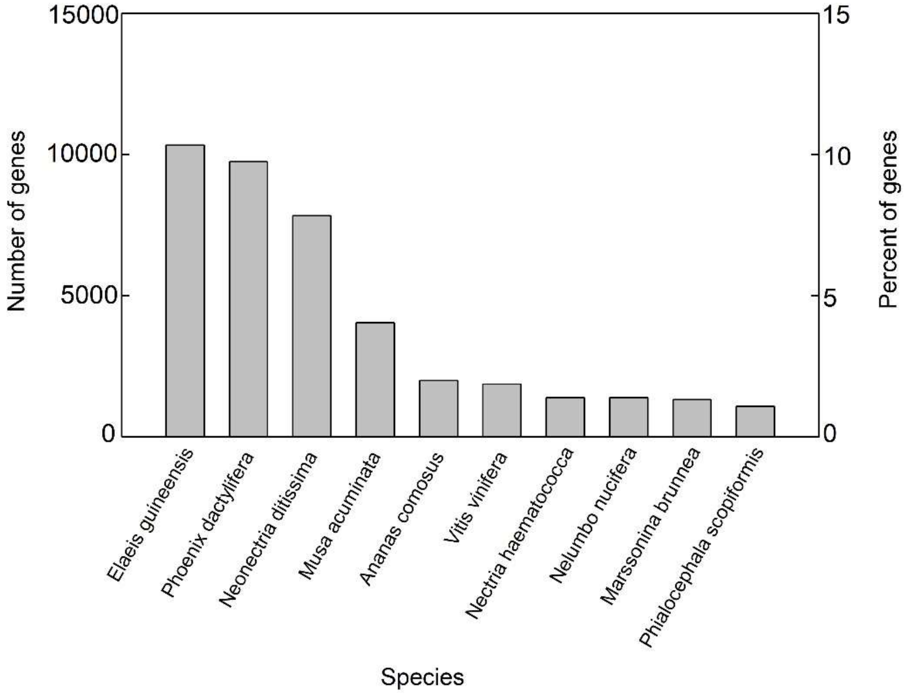

Supplementary Figure S1B. Distribution of homologous species in the Nr database matching unigenes from *V. nigrum*.

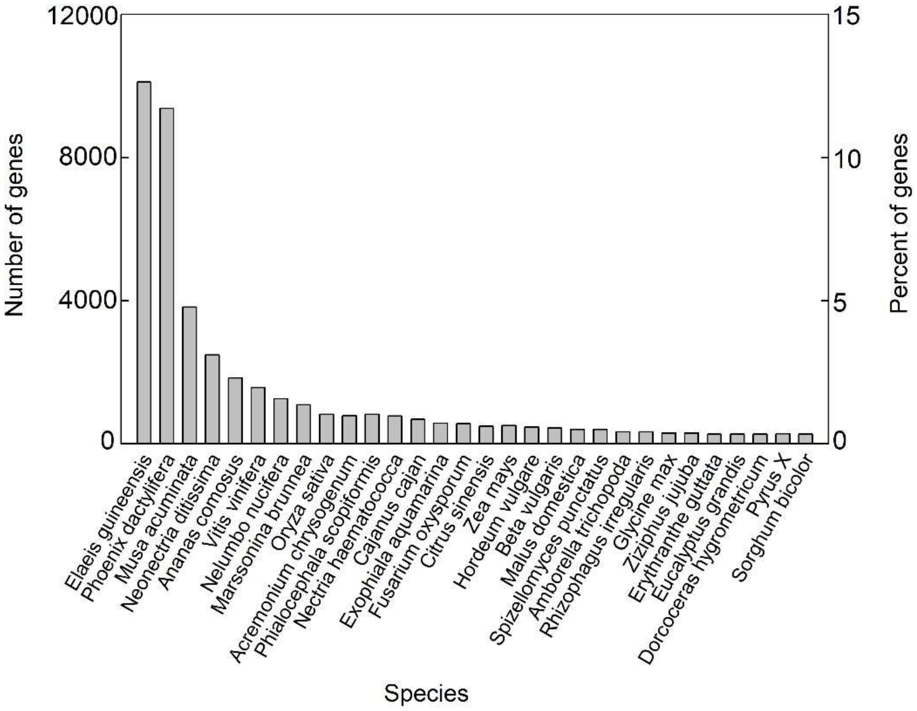

Supplementary Figure S1C. Distribution of homologous species in the Nr database matching unigenes from *V. maackii*.

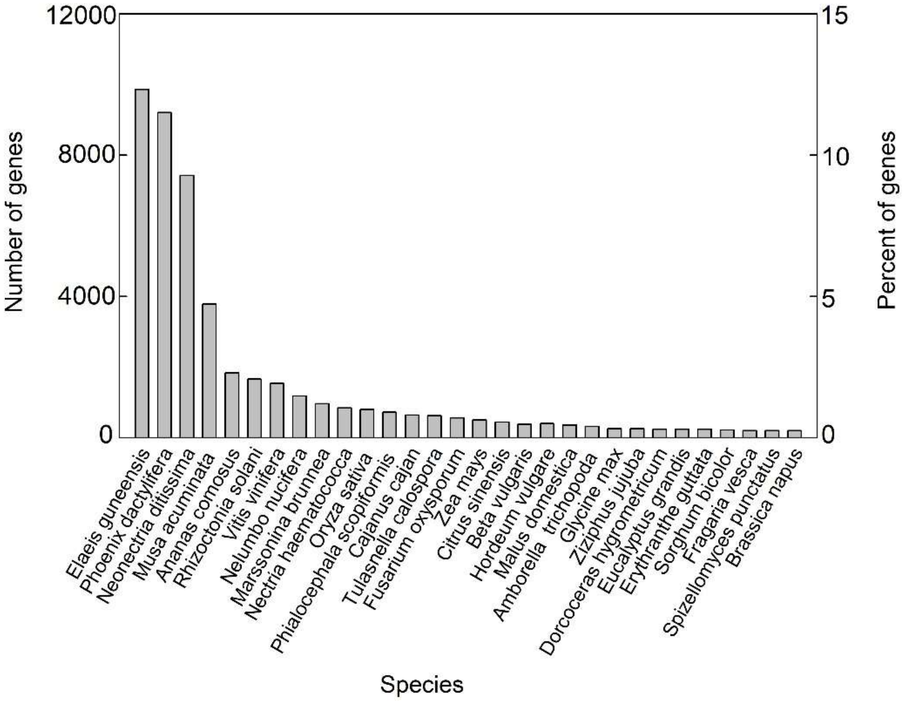

Supplementary Figure S2A. KEGG annotation of *V. maackii* and *V. nigrum* unigenes.

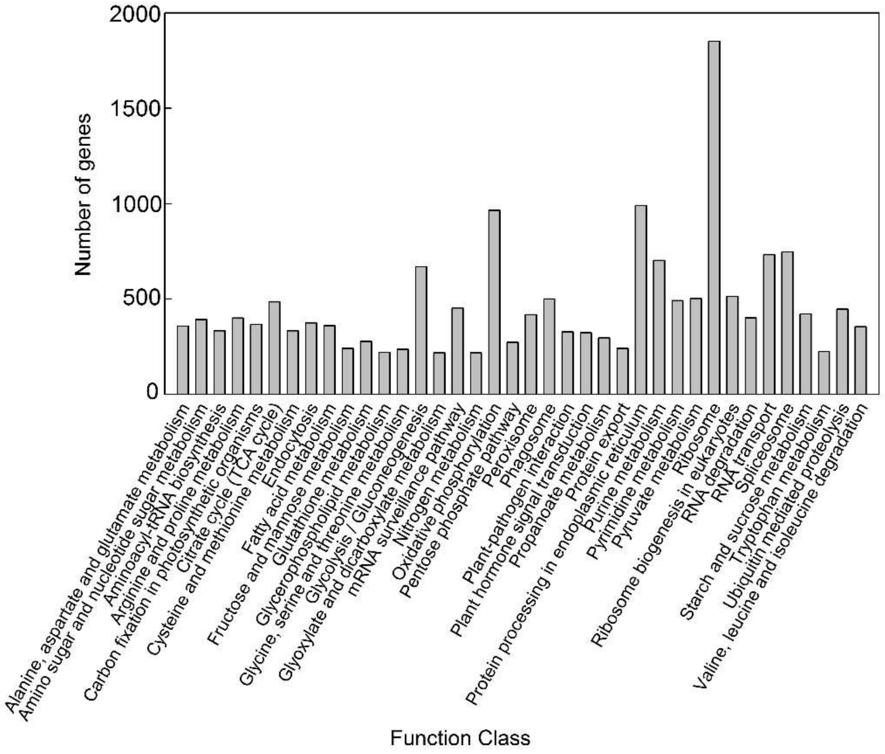

Supplementary Figure S2B. KEGG annotation of *V. nigrum* unigenes.

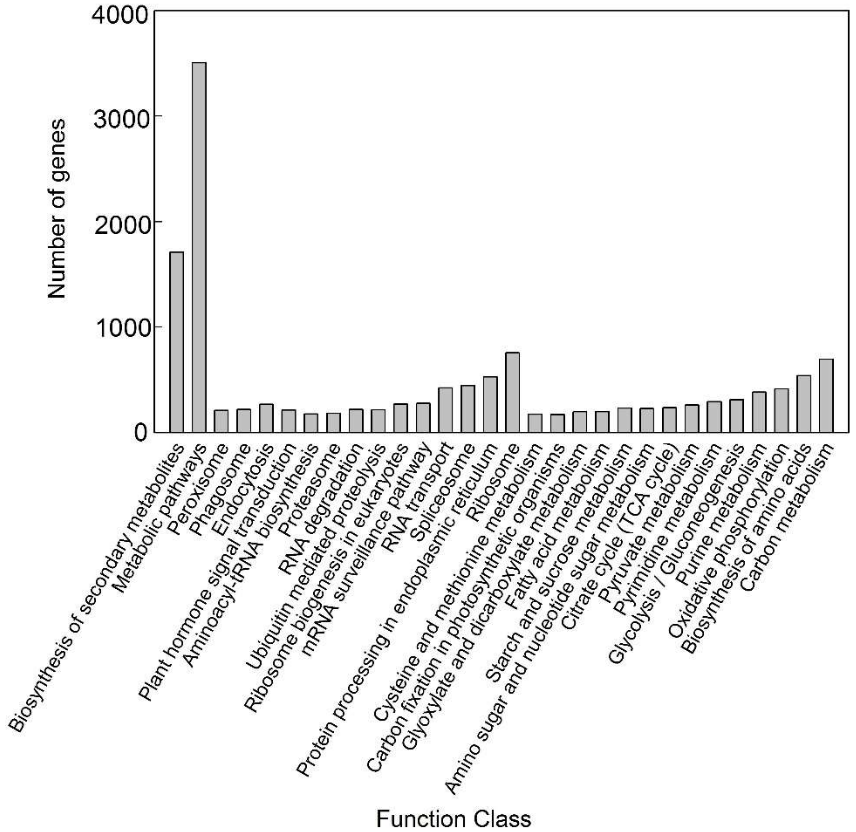

Supplementary Figure S2C. KEGG annotation of *V. maackii* unigenes.

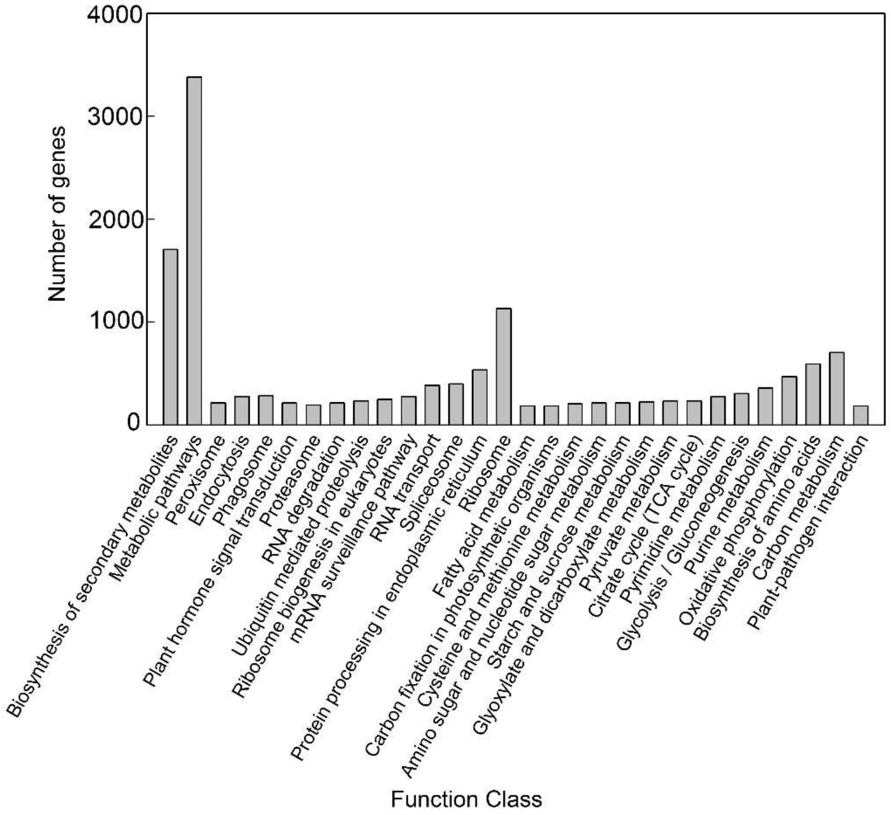

Supplementary Figure S3. Distribution of sequences among transcription factor families in (A) *V. maackii* and (B) *V. nigrum*.

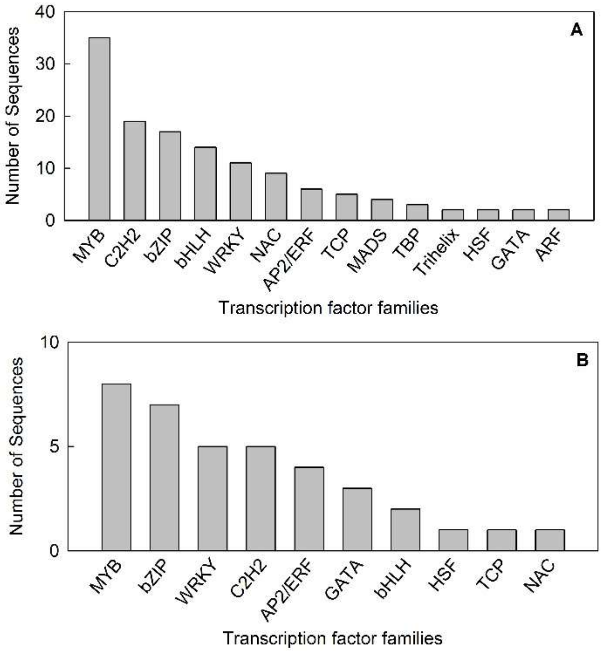

Supplementary Figure S4. Distribution of sequences among *V. maackii* and *V. nigrum* transcription factor families in (A) roots and (B) leaves.

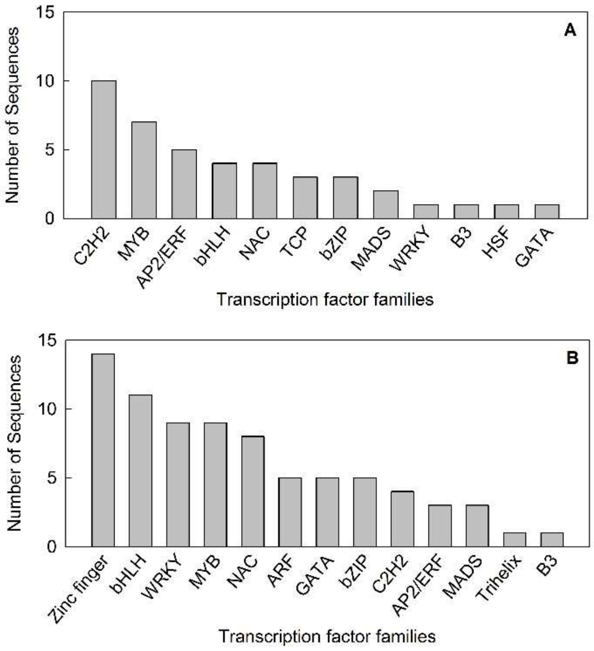

Supplementary Figure S5A. Venn diagram showing the unigene distribution in the roots and leaves of *V. nigrum*.

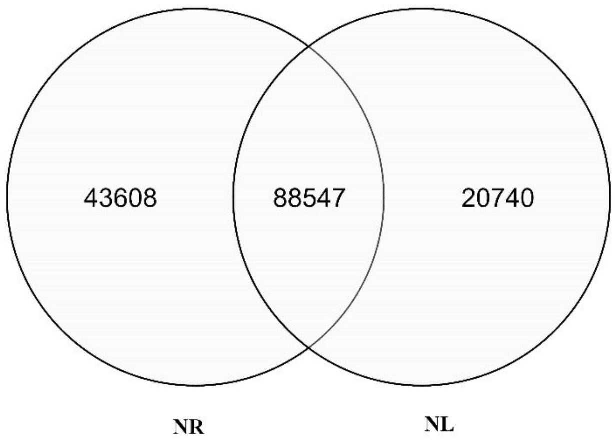

Supplementary Figure S5B. Venn diagram showing the unigene distribution in the roots and leaves of *V. maackii*.

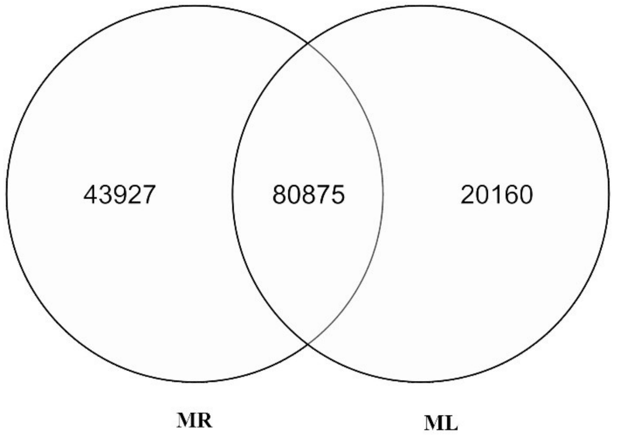

Supplement: Supplementary file 1 — Supplementary Figures. [file 41598_2023_35429_MOESM1_ESM.pdf]
